# A new screening of preterm birth in gestation with short cervix after pessary plus progesterone

**DOI:** 10.61622/rbgo/2024rbgo39i

**Published:** 2024-09-06

**Authors:** Marcelo Santucci França, Valter Lacerda de Andrade, Alan Roberto Hatanaka, Roberto Santos, Francisco Herlanio Costa Carvalho, Maria Laura Costa, Gabriela Ubeda Santucci França, Rosiane Mattar, Ben Willem Mol, Antonio Fernandes Moron, Rodolfo de Carvalho Pacagnella

**Affiliations:** 1 Escola Paulista de Medicina Universidade Federal de São Paulo São Paulo SP Brazil Escola Paulista de Medicina, Universidade Federal de São Paulo, São Paulo, SP, Brazil.; 2 Impacta Digital Academy Data Science São Paulo Brazil Impacta Digital Academy Data Science, São Paulo, Brazil.; 3 Department of Women, Children and Adolescents Health Universidade Federal do Ceará Fortaleza CE Brazil Department of Women, Children and Adolescents Health, Universidade Federal do Ceará, Fortaleza, CE, Brazil.; 4 Department of Tocogynecology, School of Medical Sciences Universidade Estadual de Campinas Campinas Sao Paulo Brazil Department of Tocogynecology, School of Medical Sciences, Universidade Estadual de Campinas, Campinas, Sao Paulo, Brazil.; 5 Department of Obstetrics and Gynaecology Monash University Clayton Victoria Australia Department of Obstetrics and Gynaecology, Monash University, Clayton, Victoria, Australia.

**Keywords:** Preterm birth, Cervix uteri, Progesterone, Logistic models, Screening, Pessaries, Ultrasonography

## Abstract

**Objective:**

This study aims to create a new screening for preterm birth < 34 weeks after gestation with a cervical length (CL) ≤ 30 mm, based on clinical, demographic, and sonographic characteristics.

**Methods:**

This is a *post hoc* analysis of a randomized clinical trial (RCT), which included pregnancies, in middle-gestation, screened with transvaginal ultrasound. After observing inclusion criteria, the patient was invited to compare pessary plus progesterone (PP) versus progesterone only (P) (1:1). The objective was to determine which variables were associated with severe preterm birth using logistic regression (LR). The area under the curve (AUC), sensitivity, specificity, and positive predictive value (PPV) and negative predictive value (NPV) were calculated for both groups after applying LR, with a false positive rate (FPR) set at 10%.

**Results:**

The RCT included 936 patients, 475 in PP and 461 in P. The LR selected: ethnics white, absence of previous curettage, previous preterm birth, singleton gestation, precocious identification of short cervix, CL < 14.7 mm, CL in curve > 21.0 mm. The AUC (CI95%), sensitivity, specificity, PPV, and PNV, with 10% of FPR, were respectively 0.978 (0.961-0.995), 83.4%, 98.1%, 83.4% and 98.1% for PP < 34 weeks; and 0.765 (0.665-0.864), 38.7%, 92.1%, 26.1% and 95.4%, for P < 28 weeks.

**Conclusion:**

Logistic regression can be effective to screen preterm birth < 34 weeks in patients in the PP Group and all pregnancies with CL ≤ 30 mm.

## Introduction

Preterm birth is the most important cause of mortality under 5.^1^ Worldwide 15 million preterm births occur, and part of them could be avoided if they were predictable.^2^ The extremely preterm birth is regularly not predicted on time to be mitigated. Cervical length is the most useful medical technique to identify the risk of preterm birth, but the low sensitivity is a characteristic of this method, with 30% of detection.^3^ New technologies must be developed to achieve the best performance in screening preterm birth, especially in severe cases < 28 weeks. These cases of extremely preterm birth are associated with cerebral palsy, enterocolitis, severe distress respiratory syndrome, leukomalacia, sepsis, and perinatal death.^4^ The costs involved are tremendous and only one preterm avoided could save around 200 thousand dollars in the first year after birth.^5^ There is no risk calculator for extremely preterm birth, and these cases are rare, but when they occur, they are very traumatic for parents and the medical system. About 1 to 3% of preterm births occur below 28 weeks, considering that 15 million cases of preterm births occur every year, about half a million of them are born < 28 weeks, which means that 100 billion dollars are spent every year, 26 billion dollars of which in the USA alone.^6,7^ The screening of preterm birth is classical and is based on a very well-done study from 1996 with sensitivity close to 30-40%, without considering severe cases or late preterm birth, but new mathematical concepts have been developed and AI algorithms has been created.^8,9^ The screening of preterm birth could be more precise with these new mathematical models and degrees instead of graduation of preterm birth could be screened.^10^ According to the series “Born too Soon” by Dean et al, 2013, it is important to develop new strategies of screening considering epidemiologic research that assesses the impact of implementing interventions and discovery science that better elucidates the complex causal pathway of preterm birth and helps to develop new screening and intervention tools.^11^The objective of this study is to identify which predictive variables can identify preterm birth < 34 weeks in high-risk women receiving treatment for preterm birth that could be useful to discriminate better treatment outcomes.

## Methods

This study is a *post hoc* analysis of a randomized clinical trial (P5 study) an open-label RCT between July 15, 2015, and March 29, 2019, executed in 17 hospitals. The P5 (Pessary Plus Progesterone to Prevent Preterm Birth) trial was submitted for registry within the Brazilian Clinical Trial Registry as UTN: U1111-1164-2636.

As a screening process, women with pregnancies (singleton or twin) between 18 0/7 and 22 6/7 weeks of gestation were invited to cervical length measurement by transvaginal ultrasonography, performed by GE Logic C5 or Samsung SW80 equipment, according to standard procedures.

Before trial initiation, all sonographers were qualified in cervical measurement according to the Fetal Medicine Foundation training program and skilled by an online training program through explanatory videos and tutorials produced by the coordinator center.

An informed consent was applied to all women before transvaginal ultrasound. For the cervical length measurement, the participant was with an empty bladder, placed in the gynecology position, and a vaginal probe was introduced into the vagina in the direction of the anterior fornix, avoiding cervices’ pressure.

The measure of cervical length was performed in a mid-sagittal view, defined as the straight-line distance between the internal and external os (straight cervical length). The curve measured inside the cervix, keeping track of the cervical gland area was defined as a curve of cervical length. Transverse and anteroposterior cervical diameters were performed. The funneling length, cervical OS dilation, size of bigger funneling, and amniotic fluid sludge were also identified.

Short cervical length was defined as a cervical length of 30 mm or less, it was the criteria to be eligible for the RCT. Women with painful contractions, preterm prelabour rupture of membranes, vaginal bleeding, placenta previa, a cerclage in situ, severe liver disease (including cholestasis), previous or current thromboembolism, cervical dilation greater than 1 cm, higher order multifetal gestation (triplets or higher), monoamniotic twin pregnancy, stillbirth of at least one fetus, and major fetal malformation were not eligible.

After written informed consent, sociodemographic, medical, and obstetric history, in addition to information about the current pregnancy were collected using a structured questionnaire. Women were randomly allocated to vaginal pessary plus vaginal progesterone (pessary plus progesterone group) or vaginal progesterone only (progesterone only group). Randomization was stratified by center, number of fetuses (one or two), and cervical length (26–30 mm or 25 mm or less) using a 1:1 ratio.

Randomization was centralized in an online database using a computer-generated algorithm. The researchers logged into the web platform (https://www.gsdoctor.com.br/Default.aspx) and had access to research forms; after including basic information and checking eligibility, they filled out a randomization form. Only after recording all information, they receive the allocation group. Due to the nature of the intervention, the study was not blinded.

Women allocated to the pessary plus progesterone group received the pessary insertion within 72 hours after the randomization. The Ingamed AM silicone pessary (unique size: outer diameter 70 mm, height 25 mm, and inner diameter 27 mm with indentations) was applied. The pessary was placed by a trained obstetrician in the outpatient clinic, with the smaller diameter placed enclosing the cervix.

The pessary was not counseled to be removed until the 36th week of gestation, except in cases of active vaginal bleeding or signs of preterm labor, premature rupture of the membranes, severe pain associated with labor, or medically indicated birth before 36 weeks of gestation. Participants in both groups received vaginal progesterone 200 mg per day until 36 weeks of gestation. Women were instructed to insert the progesterone pills into the vagina at night, immediately before sleep.

An analysis of compliance was performed for progesterone use, counting the pills in every appointment. Women in both the pessary plus progesterone and progesterone-only groups received otherwise similar obstetric care, with antenatal care according to local protocols. The perinatal data were obtained by medical records and by a national system of live-born declaration (SINASC). All possible predictor variables were considered, and we achieved 31 variables to perform the logistic regression (Chart 1). The endpoint of this study was preterm birth (before 34 weeks or before 28 weeks).

**Chart 1:** Different predictor variables obtained between 18 and 22 weeks 6/7, considered for Logistic regression

Demographic	Obstetric history	Associated diseases	Cervical characteristics
Age (years)	Number of previous miscarriages	Uterine Anomaly	Straight Cervical length (mm)
Race	Number of previous pregnancies	LBW in previous pregnancy (< 2,5kg)	Curve Cervical length (mm)
Schooling	Number of previous vaginal delivery	Chronic Diseases	Anteroposterior cervical diameter (mm)
Height (cm)	Number of previous c-sections	Comorbidities	Transversal cervical diameter (mm)
Weight (Kg)	Previous Curettage	Use of alcohol and drugs	Funneling length (mm)
Gestational age at cervical ultrasound (weeks)	Previous preterm birth	Use of Cocaine and Crack	Cervical OS dilation (mm)
Smoking	Previous cerclage	Previous cervical amputation	Size of bigger funneling (mm)
Singleton or Twin gestation			Presence of funneling
			Presence of amniotic fluid *Sludge*

In chart 1 the variables considered predictable to screen preterm birth were selected to compound logistic regression. The process of vectorization was executed on each variable to ascertain the extent of involvement of different segments within the continuous variable in cases of preterm birth occurring before 34 weeks within the pessary plus progesterone group (n=475). Once the optimal variable was identified, three distinct cohorts were selected for logistic regression validation:

1. Patients within the pessary plus progesterone group (n=475) with cervical length < 15 mm.
2. Patients in the Progesterone-only group (n=461) experienced preterm birth before 34 weeks.
3. Patients in the Progesterone-only group (n=461) experienced preterm birth before 28 weeks.

The statistical analysis was performed by SPSS 23.0 (SPSS Inc., Chicago, IL), and SQL Management studio and Jupiter. Significance was established by p < .05, two-sided.

A stepwise backward logistic regression was performed, the inclusion/exclusion criteria were p< 0.05, and clinical aspects were evaluated to decide how variables will be included in the final regression. The best variable, with the most statistically significant variables, was included. The ROC curve, AUC (CI95%), sensitivity, specificity, PPV, and PNV were performed after a fixed 10 and 20% of false-positive rate (FPR), which determined the cutoffs for the measures. The rationale for determining the cutoff of sensitivity and specificity was the false positive rates of 10% or 20%, usually applied in the literature.^12^ For a simple comparison of the performance, we created a ROC curve in the same Group (pessary plus progesterone) of the cervical length < 15 mm.

A validation with Progesterone only group was performed by applying the logistic regression coefficient looking for the most severe cases. An analysis with the target in preterm birth < 34 weeks and < 28 weeks was performed to achieve the best ROC curve in the Progesterone group. AUC (CI95%), sensitivity, specificity, PPV, and PNV were calculated after a fixed 10 and 20% of the FPR.

A final analysis was performed with both groups together considering the best performance validation and sensitivity, specificity, PPV, and PNV were calculated for the Pessary plus progesterone group < 34 weeks and for the Progesterone group with the best performance, fixed 10 and 20% of the FPR. The PECEP clinical trial projects a 22% composite poor neonatal outcome rate in women with a short cervix^13^ and deems a 10% reduction clinically significant; to detect a decrease from 22% to 12%, 438 women (219 per group) are suggested for randomization. Additionally, a subgroup analysis focusing on cervical length ≤25 mm proposes a sample increase to 468 women (234 per subgroup) to achieve a 50% reduction in adverse neonatal outcomes, resulting in a final recommended sample size of 936 women for the study.^14^

The National Research Ethics approved the protocol under number 38417114.0.1001.5404 and each center received protocol approval through their local Institutional Review Boards. After approval for the national board, the local Board of Ethics was also approved under number 38417114.0.2007.5505.

## Results

**Figure 1:**
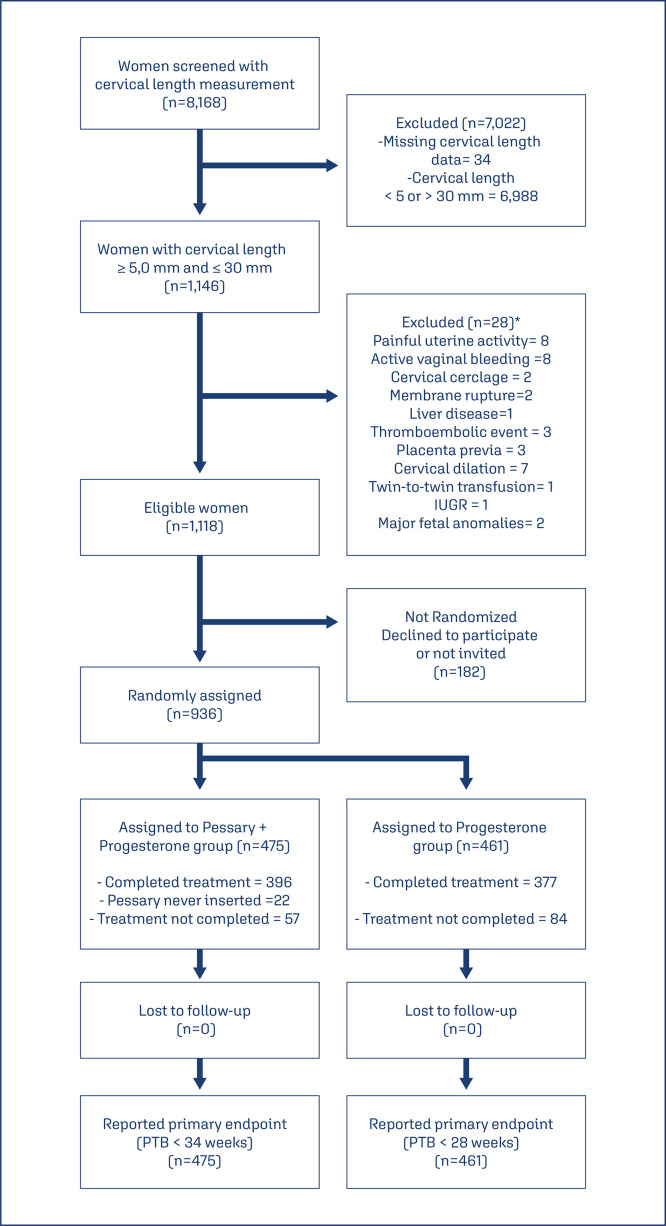
Patient selection process

The screening was performed on 8,168 pregnant women, 1,146 were eligible with cervical length ≤ 30 mm, obeying the inclusion criteria in P5 Study 936 gestation, which were randomized (1:1) (Figure 1), and demographic characteristics of P5 study were expressed in Table 1 - supplement. After the end of the study, 31 variables were chosen as predictors of preterm birth, and a vectorization was applied to create dummy variables. One hundred and thirty-one dummies variables were created and inserted into stepwise backward logistic regression (LR) and the relative risk was calculated for each dummy (Table 1). The most relevant variables presented by LR were ethnics white [OR 2.532 (CI95% 1.164-5.508); p = 0.019], absence of previous curettage [OR 0.113 (CI95% 0.049-0.258); p< 0.0001], singleton gestation [OR 0.135 (CI95% 0.052-0.349); p< 0.0001], short cervical length included < 19 weeks’ gestation [OR 3.373 (CI95% 1.379-8.248) p =0.008], straight-line cervical length < 14.7 mm [OR 4.072 (CI95% 1.505-11.006);p=0.006], cervical length in curve > 21.0 mm [OR 0.216 (CI95% 0.094-0.498); p<0.0001]. The inclusion of one variable was performed by entering mode to obtain the best clinical logistic regression with the inclusion of previous preterm birth < 37 weeks [OR 3.647 (CI 95% 1.650-8.058); p =0.001] in the results (Table 2).

**Table 1:** Demographic characteristics between pessary plus progesterone group and progesterone only group, for P5 study

Basic characteristics	Pessary + Progesterone Group (n=475)	Progesterone Group (n= 461)
Ethnics		
White	166(34.9)	159(34.5)
Black	63(13.3)	68(14.7)
Mixed	244(51.4)	228(49.5)
East Asian	2(0.4)	3(0.6)
Other	0(0)	2(0.4)
Maternal Age	26.5 ± 7.0	26.3 ± 6.6
BMI	25.9 ± 5.2	26.0 ± 5.6
Schooling (Years)		
Elementary School	113(23.8)	96(20.8)
Middle School	307(64.6)	304(65.9)
High School and University	55(11.6)	61(13.2)
		
Marital Status		
Living with a partner	376(79.2)	370(80.3)
Not living with a partner	99(20.8)	91(19.7)
Number of previous pregnancies		
0	192(40.4)	197(42.7)
1	122(25.7)	123(26.7)
2 or more	161(33.9)	141(30.6)
Previous vaginal delivery	181(38.1)	170(36.9)
Previous C-section delivery	60(12.6)	63(13.7)
Previous preterm birth	91(19.2)	85(18.4)
Previous miscarriage	143(30.1)	128(27.8)
Previous curettage	85(17.9)	72(15.6)
Chronic diseases	80(16.8)	71(15.5)
Previous cervical amputation	11(2.3)	16(3.5)
Uterine anomaly	11(2.3)	9(2.0)
Previous cerclage	5(1.1)	5(1.1)
Type of Pregnancy		
Singleton	432(90.9)	433(93.9)
Twins	43(9.1)	28(6.1)
Conception method		
Spontaneous	470(98.9)	457(99.1)
Assisted reproduction techniques	5(1.1)	4(0.9)
Cervical Length at randomization		
>25 mm	215(45.3)	212(46.0)
≤ 25 mm	260(54.7)	249(54.0)
*Sludge* at randomization	73(15.4)	70(15.2)
Funneling at randomization	110(23.2)	115(24.9)
Gestational age at randomization		
18	51(10.7)	45(9.8)
19	61(12.8)	67(14.5)
20	83(17.5)	95(20.6)
21	124(26.1)	109(23.6)
22	156(32.8)	145 (31.5)

**Table 2:** Principal variables selected by Logistic Regression to predict outcomes in the treatment groups

	Coef Beta	E.P.	Wald	Sig.	Odds ratio	95% C.I. para EXP(B)	
						Inferior	Superior
Ethnics white	0.929	0.396	5.492	0.019	2.532	1.164	5.508
Absence of previous curettage	-2.182	0.421	26.852	0.000	0.113	0.049	0.258
Previous preterm birth (< 37 weeks)	1.294	0.405	10.228	0.001	3.647	1.650	8.058
Singleton gestation	-2.005	0.486	17.013	0.000	0.135	0.052	0.349
Gestational age < 19 weeks during cervical ultrasound	1.216	0.456	7.100	0.008	3.373	1.379	8.248
Straight cervical length between 5,2 and 14,7mm	1.404	0.507	7.660	0.006	4.072	1.506	11.006
Curve cervical length > 21 mm	-1.533	0.426	12.928	0.000	0.216	0.094	0.498
Constant	0.724	0.594	1.486	0.223	2.062		

Table 2 presents the most significant dummies variables to detect preterm birth < 34 weeks in Pessary plus progesterone group (n=475). Fixing 10% and 20% of the FPR, the performance to predict preterm birth of Logistic regression for the Pessary plus Progesterone Group < 34 weeks, the Progesterone Group < 28 weeks (plus perinatal death), and for all women included, were expressed in table 3.

**Table 3:** Performance of Logistic Regression approach for the detection of preterm birth for each treatment group, fixed 10% and 20% of false-positive rate

		AUC	Sensitivity		Specificity		PPV/ PNV				Real False Positive rate	
												
Estimated false positive rate			10%	20%	10%	20%	10%	20%	10%	20%	10%	20%
Pessary+ Progesterone (n=475)	LR PP <34w	0.978	83.33	93.75	98.13	88.76	83.33	48.39	98.13	99.21	2%	11%
	CL< 15 PP< 34 w	0.311	37.50	50.00	92.04	83.37	34.62	25.26	92.91	93.68	8%	17%
Progesterone (n=461)	P <34 w	0.695	26.39	43.06	93.06	84.32	41.30	33.70	87.23	88.89	7%	16%
	P < 28 w	0.765	38.71	54.84	92.09	82.56	26.09	18.48	95.42	96.21	8%	17%
All included women (n=936)	PP<34 w + P< 28 w	-	65.82	78.48	85.53	95.10	33.33	55.32	96.79	97.73	5%	14%

**Figure 2:**
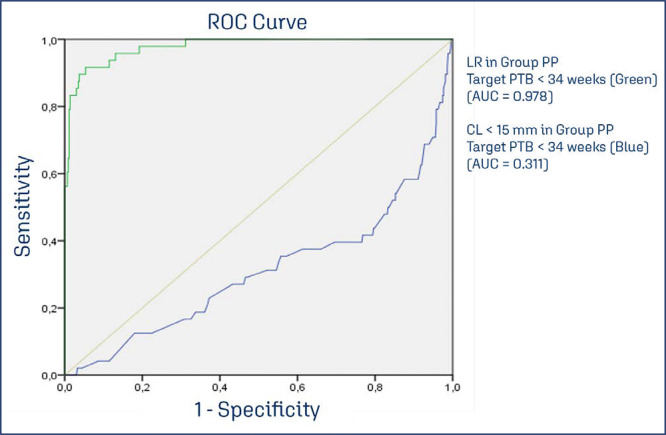
ROC curve of Logistic Regression and Cervical length< 15 mm (Group PP) targeting preterm birth < 34

The ROC curve was created and identified an AUC = 0.978 (CI95% 0.961 - 0.995), for LR, and AUC = 0.311 (CI95% 0.221- 0.402) for CL< 15 mm with a target of preterm birth < 34 weeks, for both. The performance of cervical length < 15 mm in the detection of PTB < 34 weeks confirms that LR is better screening for pessary + progesterone detection of PTB < 34 weeks. The validation of LR in the Progesterone Group performs differed for PTB at < 34 weeks and < 28 weeks. The AUC ROC curve for the Progesterone Group < 34 weeks was 0.694 (CI95% 0.622 - 0.767), and < 28 weeks (birth and perinatal death) was 0.765 (CI95% 0.665 - 0.864), Thus, the most severe PTB cases in the Progesterone Group occurs below 28 weeks and are highly associated with perinatal death (20/31). In the PP Group, this association, in contrast, was less observed (8/48). Figure 2 shows the ROC curve for Pessary plus Progesterone, CL < 15 mm, Progesterone Group with target < 34 weeks. Figure 2 shows the ROC curve for the Progesterone Group with a target in preterm birth < 34 weeks and < 28 weeks plus perinatal death.

**Figure 3:**
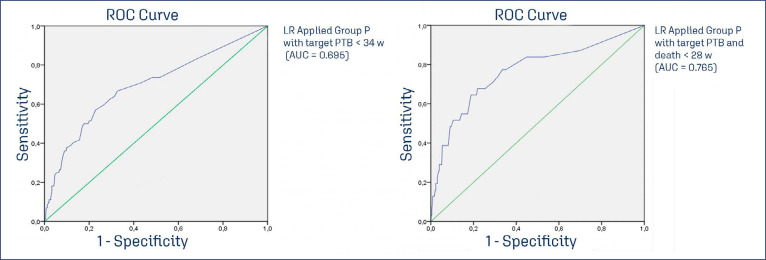
ROC curves of Logistic Regression for preterm birth < 34 weeks (left) and preterm birth and perinatal death < 28 weeks (right) in the Progesterone Group

Figure 3 presents the ROC curve, by Logistic regression (Green) and Cervical Length < 15 mm (blue) as predictors isolated of preterm birth < 34 weeks in the Pessary plus progesterone Group. This graphic is a comparison between Logistic Regression and the most important isolated variable to discriminate preterm birth (short cervix), demonstrating that Logistic Regression perform better than cervical length < 15 mm, to predict preterm birth < 34 weeks, in this study. Figure 3 presents different ROC curves with similar logistic regression applied for different targets. On the left, the LR was applied to Progesterone Group with the target in PTB < 34 weeks (AUC = 0.695), on the right, a similar LR was applied to Progesterone Group with the target in PTB < 28 weeks (AUC = 0.765), this data suggests that the most severe cases appear earlier in Progesterone Group than in Pessary plus Progesterone Group which performed better at the later period of gestation (AUC = 0.978), considering target in PTB < 34 weeks.

## Discussion

We created a new model to predict severe cases of preterm birth among high-risk women treated for preterm birth prevention, and these cases must be predicted due to their impact on health costs and infant morbidity and mortality.

Currently, all the decisions for the management of preterm birth are based on ultrasound cervical length measures. Some calculators for preterm birth are available to screen preterm birth, but these calculators are not useful in clinical practice.^15,16^ Creating easy algorithms to predict preterm birth is important because the costs involved are tremendous.^7^ Strategies joining cervical ultrasound, clinical aspects, and demographic characteristics are strongly encouraged by experts.^17^

The present study was retrospective and was a *post hoc* analysis of prospective data collected. This paper was not previously conceived for the prospective study. The objectives were conceived after the end of data collection.

In this study, a total of 475 pregnant women who were randomly assigned to receive pessary plus progesterone were assessed. The multicentric nature of the trial is noteworthy due to its substantial number of pregnancies, ensuring a comparable distribution across both groups. This approach effectively mitigates any demographic bias and significantly reinforces the validity of the findings.

The validation was performed in a similar population (Progesterone group) and the same population with another dependent variable (CL< 15 mm). The screening with CL< 15 mm showed that LR has a large advantage for the same population.

The validation of Logistic Regression applied to the Progesterone Group demonstrated the ability to identify some of the most severe cases of preterm births <34 weeks, as 26% (19/72) of cases were identified in the Progesterone Group with this target of PTB <34 weeks; however, this low sensitivity resulted in a low AUC = 0.675.

The aspects of the severity of the Progesterone cases were hidden when the analysis was performed with the target of preterm birth < 34 weeks. So, we focused on preterm birth < 28 weeks and perinatal death < 28 weeks. With this new target, we obtained AUC = 0.765 with 38% of detection of the severe cases with a false-positive rate of 10% and 54.8% of sensitivity with 20% of FPR.

This mathematical aspect can support the strong evidence that pessary plus progesterone increases the final gestational age independently of the aspects involved in pre-insertion, such as twin or singleton gestation, cervical length, or demographic characteristics.

We can observe that in the PP Group, we obtained 47 cases of preterm birth <34 weeks with only 8 perinatal deaths (8/47); in the Progesterone Group targeting preterm birth <34 weeks, we identified 19 cases by Logistic Regression (sensitivity = 26%) out of a total of 72 preterm births with a 10% False Positive Rate (FPR) resulting in an Area Under the Curve (AUC) of 0.675, associated with 20 perinatal deaths (20/72); and, in the same Progesterone Group, there were 31 cases of preterm birth <28 weeks, with 12 cases detected by Logistic Regression (sensitivity = 38.7%) with 10% FPR (AUC = 0.765) and a notably high number of 20 perinatal deaths (20/38).

Therefore, it is feasible to develop a novel calculator exclusively designed for identifying such critical instances. This tool could be utilized for advancing fresh research involving cerclage or cerclage + pessary techniques, aiming to mitigate the described unfavorable outcome accompanied by a significant loss of life.

The purpose of the P5 Study was to illustrate the variation in composite neonatal outcomes among pregnancies with cervical lengths of ≤ 30 mm.^18,19^However, for this specific target, statistically significant differences were not evident. In contrast, the present study focused on preterm birth < 34 weeks and perinatal death, revealing a more distinct contrast in outcomes between the Pessary plus Progesterone (PP) and Progesterone (P) Groups.

Within the scope of the P5 Study, roughly 200 cases with composite neonatal outcomes were evident in both study groups, in all gestational age of birth, even after 37 weeks. However, when concentrating on the subgroup of less than 34 weeks gestation, the involvement was limited to no more than 120 cases. These findings indicate that out of the total cases studied, 80 fetuses exhibited composite neonatal outcomes but presents more than 34 weeks. Thus, in P5, besides the majority of cases with composite neonatal outcome occurred in PTB < 34 weeks subgroup, it presents 40% of cases with composite neonatal outcome in fetuses without severe preterm birth, which could demonstrate CNO in Brazil do not represent preterm birth.

It is plausible that other factors contributing to composite neonatal outcomes were implicated in the P5 Study, beyond the spectrum of preterm birth. This is particularly relevant considering Brazil’s economic circumstances, and the study’s implementation within maternity settings beset by challenges typical of economically disadvantaged nations. These challenges are reflected in the composition of cases within the composite neonatal outcomes category, accounting for 21.1% (198 out of 936 total cases), which is comparable to rate of PTB < 37 weeks’ gestation (282 cases or 30.1%).

When juxtaposed with the PECEP Study conducted by Goya et al. in 2012, the prevalence of cases resulting in composite neonatal outcomes in P5 study appears notably higher, 21.1% and 9.2% (35/380 cases) in PECEP.^13^ In terms of the rate of preterm birth before 28 weeks (5.2% or 20/380 cases), the numbers are similar to the numbers of composite neonatal outcome (9.2% or 35/380) in PECEP. Comparatively, in P5 Study the incidence of adverse perinatal outcomes for the entire study (21.1% or 198/936 cases) was very different from the incidence of preterm birth < 28 weeks (3.7% or 35/936 cases).^19^

While Goya’s study perhaps more accurately captures the relationship between severe PTB and composite neonatal outcomes, the same may not be as true for P5 study conducted in Brazil. It is conceivable that preterm birth before 28 weeks may not be the most suitable parameter in our context to effectively underscore the risk of composite adverse outcomes for preterm births in our country.

The present study was conceived with mathematical methods based on machine learning, including vectorization. The vectorization probably is responsible for the good adjustment of the dummies variables into the LR. The high significance of each variable made this model a candidate to change the premise that calculators are not used in preterm birth.

So this study is very helpful to discriminate preterm birth < 34 weeks in the Pessary plus progesterone Group, with sensitivity higher than the best studies with cervical length isolated, the Logistic Regression with demographic and historical aspects associated to aspects of transvaginal ultrasound of cervix performed more than 83% of sensitivity.^20,21^

With the validation of LR in the Progesterone Group, it could also be useful to other populations without using a pessary. The performance of sensitivity in the Progesterone Group was better than cervical length isolated, presenting 54.8% with 20% of FPR. In addition, probably LR would be helpful also for non-treated.

The sensitivity of Preterm birth < 34 weeks from PP Group added to preterm birth and perinatal death < 28 weeks from P Group, with 80% of sensitivity, and 85% of specificity with 20% of FPR, demonstrates that the extreme and very preterm could be detected by this LR in gestations with CL ≤ 30 mm. And it must be highlighted that these severe cases occur despite treatment. In the PP Group, there are fewer fatal cases than in the Progesterone Group, but a total of 28 fetal perinatal deaths were registered in this study associated with preterm birth, in 119 births < 34 weeks (23.5%) despite the treatment.

So, the validation in the Progesterone group presents an aspect not expected in the initial project. There was a possibility to believe that this logistic regression can be a good predictor of preterm birth for all populations with cervical length ≤ 30 mm, treated or not with pessary or progesterone, with an emphasis on the detection of extremely and very preterm birth.

In other words, the logistic regression applied as a calculator to screen PTB < 34 weeks has great potential. This is due to its higher sensitivity (80%) to detect PTB in pregnant women with cervical length ≤ 30 mm if compared to the current screening method based exclusively on cervical length (≤ 25 mm), which presents around 30% of sensitivity, without increasing the real false positive rate among this different techniques (10% to 14%).^21^However, a prospective study using this new calculator must be conceived to confirm this theoretical performance.

## Conclusion

The logistic regression algorithm can be an important tool to identify patients with a high risk for preterm birth < 34 weeks that receive treatment for preterm birth prevention and for all patients with cervical length ≤ 30 mm. It increases the sensitivity for the vast majority of patients with a risk of PTB, without increasing the current false positive rate.

Logistic regression was conducted for the pessary group combined with progesterone, targeting preterm birth < 34 weeks (LR PP<34w). The same logistic regression was applied within the Progesterone Group targeting preterm birth < 34 weeks (P<34w) and < 28 weeks (P<28w). As a metric, a simple comparison was performed to assess the performance of a short cervix < 15 mm in the Group Pessary + progesterone for detecting preterm birth < 34 weeks (CL<15 PP<34w); PP-Pessary plus progesterone group; P- Progesterone only group
